# Assessment of quality of alcohol-based hand sanitizers used in Johannesburg area during the CoViD‐19 pandemic

**DOI:** 10.1038/s41598-022-08117-z

**Published:** 2022-03-10

**Authors:** Puleng Matatiele, Bianca Southon, Boitumelo Dabula, Talulani Marageni, Poobalan Poongavanum, Boitumelo Kgarebe

**Affiliations:** Analytical Services, National Institute for Occupational Health, National Health Laboratory Service, P.O. Box 4788, Johannesburg, South Africa

**Keywords:** Biochemistry, Health care, Chemistry

## Abstract

Since the outbreak of the Coronavirus Disease 2019 (CoViD-19), the World Health Organization has recommended that, in absence of soap and water, alcohol-based hand sanitizer can be used to prevent the transmission of coronaviruses. Unfortunately, many media and anecdotal reports indicate that many alcohol-based hand sanitizers sold in South Africa are substandard and some contain potentially toxic ingredients. The study aimed to identify hand sanitizers used in the Johannesburg area during the CoViD-19 pandemic that do not contain the recommended alcohol concentration of at least 70% propanol or 60% ethanol, and contain traces of toxic ingredients. Hand sanitizers randomly collected from various traders around Johannesburg were analyzed using Agilent auto sampler coupled to a gas chromatograph utilizing flame ionisation detection. Of the 94 hand sanitizer samples collected, three preparations contained no alcohol, whereas the rest contained either ethanol, 2-propanol or 1-propanol or a combination of two alcohols. Of the alcohol-containing hand sanitizers, 37 (41%) contained less than 60% alcohol. Ethyl acetate, isobutanol and other non-recommended alcohols (methanol and 3-methyl-butanol) were also identified. Consumers are therefore warned that among the many brands of hand sanitizers found around Johannesburg, there are some substandard preparations and some that contain traces of toxic ingredients.

## Introduction

The gold standard for hand hygiene and prevention of the spread of non-airborne infectious diseases is regarded as washing with warm water and soap, because water and soap remove oils from hands that can harbour pathogens^1^. However, in the absence of water, hand sanitizers are recommended^2,3^. The transmission of respiratory pathogens spread by droplet or airborne routes is limited through respiratory hygiene/cough etiquette and physical space infection prevention measures^4,5^.

Since the outbreak of SARS-CoV-2, CoViD-19 (coronavirus), it is recommended by the World Health Organisation (WHO) that, in absence of water, the use of alcohol-based hand sanitizers can prevent the transmission of coronavirus^6^. Consequently, the demand for hand sanitizers has increased worldwide including South Africa, resulting in a surge in the trade of hand sanitizers and initially leading to shortages in their supply.

Hand sanitizer formulations exist in the form of liquids, gels and foams. Depending on the active ingredient used, hand sanitizers can be classified as one of two types: alcohol-based and alcohol-free. Alcohol-based hand sanitizers are recommended for general use, whereas the alcohol-free ones are not^7,8^. Hand sanitizers with less than the recommended alcohol content (60–95% alcohol) have been found not to work well for many types of pathogens, in that they may merely reduce their growth rate and hence reduce their numbers rather than kill them outright^9,10^.

Alcohol-based hand sanitizers are available in the form of rinses (liquid) and rubs (gel, foam and cream), and both are effective agents for reducing the number of viable pathogens, including coronavirus, on hands. Alcohol-based hand sanitizers may contain a variety of alcohols [e.g., isopropyl alcohol (isopropanol, 2-propanol), ethanol (ethyl alcohol), *n*-propanol (1-propanol)] or a combination of two of these, including other ingredients^11–14^.

For alcohol-based hand sanitizers, the US Centres for Disease Control and Prevention (CDC) recommends a concentration of 60–95% ethanol or 2-propanol mixed with distilled water^15^. Alcohol acts on microbes in the presence of water by making the organism cell membrane permeable leading to cytoplasm leakage, denaturing of proteins and eventually, cell lysis^12^. At higher concentrations (> 95%) alcohol is not effective since microbial denaturing of proteins only takes place in the presence of water^16^. Alcohols with four carbons and more are hence, not recommended to be used as hand sanitizers since they are less soluble in water^2^.

Ethanol has been shown to be effective against a variety of enveloped viruses, beginning at concentrations of 42.6%^17^. Addition of acids to ethanol can substantially improve the virucidal activity against most viruses^17^. For example, a formulation with low alcohol content and citric acid was found to inactivate all enveloped and non-enveloped viruses^18^. Several studies demonstrate that 2-propanol is considerably less effective compared to ethanol against viruses^17^. Some studies have also shown that ethanol gel formulations, unless they have been specially formulated and tested are less efficacious than ethanol solution formulations^19^, even though this has not yet been proven for SARS-CoV-2.

As previously indicated the global medical crisis as a result of the CoViD-19 pandemic has resulted in a great surge in the trade of hand sanitization products. This emergent situation is expected to continue for a considerable period of time until more efficient infection preventive measures become available, hence hand sanitizer demand will remain for an extended time. Unfortunately, many hand sanitizers in South Africa have not been verified to meet the regulators’ recommendations or that they are manufactured under the stipulated regulatory conditions^20,21^. In addition, the regulator [South African Bureau of Standards (SABS)] lacks verifiable information to ascertain the methods being used to prepare hand sanitizers at homes and to determine if these sanitizers are safe for use on human skin. As part of public awareness campaign and contribution to assist during the CoViD-19 pandemic, the project aimed to identify sanitizers available and used in the Johannesburg area that do not contain the recommended quality and alcohol content. In South Africa, alcohol-based hand sanitizers must comply with the standard SANS 490 as recommended by SABS^20^. The standard specifies that a minimum of 70% alcohol content is required if; alcohol, such as ethanol, isopropanol or *n*-propanol is the main ingredient; and that 60% alcohol content is required if there are other active ingredients. Solvents such as acetone (propanone), methanol, methylated spirits or other spirits are not allowed to be used.

## Materials and methods

### Collection of hand sanitizer samples

Ninety-four (94) samples of hand sanitizer sold in retail stores, spaza shops (informal convenience shop business in South Africa) and by street vendors, were randomly collected around Johannesburg during the period March to June 2020. The products were purchased ensuring not to buy repeat products/brands. Where two products of the same brand were included in the study it was so that one represents a gel and the other a liquid hand sanitizer. The hand sanitizer (HS) samples were labelled as HS1 to HS94 (Table 1).

**Table 1:** A list of hand sanitizers whose alcohol content was assessed.

Code	Trade name	Gel/liquid	% Alcohol (stated on container)
HS 1	ADCO HYGIENE (HD)	Liquid	70
HS 2	ADCO HYGIENE (WG)	Gel	40
HS 3	Alcosan	Liquid	Not stated
HS 4	Alcosan L (FG)	Aerosol	40
HS 5	Ackermans	Gel	Not stated
HS 6	BIOSOL	Gel	70
HS 7	Century Chemicals	Gel	Not stated
HS 8	Chaitoo Medical Supplies	Liquid	70
HS 9	Classic Guard	Gel	70
HS 10	Classic Guard	Liquid	70
HS 11	Zamtha Hygiene	Gel	70
HS 12	Clere	Gel	Not stated
HS 13	Clere	Gel	Not stated
HS 14	Clicks Expert	Gel	70
HS 15	Cliks Helping Hand Trust	Gel	Not stated
HS 16	Cosmo essentials	Gel	63
HS 17	Cuticura	Gel	Not stated
HS 18	Devlon	Liquid	74
HS 19	DH	Gel	70
HS 20	No Name	Gel	Not stated
HS 21	dpachem	Liquid	70
HS 22	ef-active	Gel	72
HS 23	Garnier	Gel	65
HS 24	Germ Bros	Liquid	95
HS 25	Germ-xterminator	Gel	Not stated
HS 26	GERMEX	Gel	Not stated
HS 27	Lemon Verbena	Gel	Not stated
HS 28	Handi Kleen	Gel	Not stated
HS 29	HiDerm	Liquid	70
HS 30	Hydralab	Gel	70
HS 31	Identity	Gel	65
HS 32	Identity	Gel	Not stated
HS 33	i-Med	Aerosol	70
HS 34	IMPO	Liquid	70
HS 35	Izemo Services Group	Liquid	Not stated
HS 36	Journey	Aerosol	62
HS 37	Kmanufacturing	Liquid	70
HS 38	Laboratoire Armille (Fyto)	Liquid	70
HS 39	Lifebuoy	Gel	Not stated
HS 40	Liquid Clinic	Liquid	70
HS 41	Liquid Clinic	Aerosol	70
HS 42	Little Animals	Liquid	Not stated
HS 43	LP	Gel	71
HS 44	Mellow	Gel	62
HS 45	Micro Safe	Gel	70
HS 46	Milton	Gel	Not stated
HS 47	Nature's Nourishment	Gel	62
HS 48	No Germ	Liquid	70
HS 49	No Name	Liquid	70
HS 50	No Name	Liquid	Not stated
HS 51	No Name	Liquid	70
HS 52	No Name	Liquid	Not stated
HS 53	No Name	Gel	Not stated
HS 54	No Name	Gel	Not stated
HS 55	No Name	Liquid	70
HS 56	Noxaderm	Gel	70
HS 57	Oh So Heavenly	Gel	Not stated
HS 58	Omni Protect	Aerosol	70
HS 59	Pakmed	Liquid	70
HS 60	Pepper Tree	Gel	Not stated
HS 61	Phepha Hand Cleansing Sanitizer	Liquid	72
HS 62	ProCare	Gel	70
HS 63	Puresse	Gel	Alcohol Free
HS 64	Puridene	Liquid	60
HS 65	Puridene	Liquid	70
HS 66	Renew	Gel	70
HS 67	Ryadsa Med (Lavender Blue)	Liquid	Not stated
HS 68	SA Chemical Products (Hy-gene)	Liquid	Not stated
HS 69	Safeguard	Gel	Not stated
HS 70	Sani	Gel	70
HS 71	SHARP	Liquid	70
HS 72	Soft Chemical Laboratories	Aerosol	70
HS 73	Triple K	Liquid	75
HS 74	Unicad cleaning solutions (Eco Blast)	Aerosol	Not stated
HS 75	Voi (pamper yourself)	Gel	Not stated
HS 76	VP Herbal	Gel	70
HS 77	Woolworths Food Hand Cleanser	Gel	Not stated
HS 78	Woolworths Food Hand Cleanser (Pineapple)	Liquid	Not stated
HS 79	Woolworths Food Hand Cleanser (spray cucumber)	Liquid	Not stated
HS 80	Hand San Plus	Gel	75
HS 81	Manly	Aerosol	70
HS 82	Scarlet Hill (your hands biggest fan)	Gel	70
HS 83	Life Trek	Liquid	75
HS 84	No Name (Mr Price)	Liquid	Not stated
HS 85	Paw Patrol	Gel	Not stated
HS 86	Pride (Xtra Care)	Gel	Not stated
HS 87	Sani Hand sanitiser (Blue)	Gel	Not stated
HS 88	Bennetts Family Care	Aerosol	80
HS 89	Clean	Gel	70
HS 90	ProCare	Gel	Not stated
HS 91	No Name	Liquid	Not stated
HS 92	W.LAB	Gel	70
HS 93	NIOH Sample	Liquid	Not stated
HS 94	No Name	Liquid	Not stated

### Preparation of internal standard (2% acetaldehyde)

Two millilitres (2 ml) of acetaldehyde (Sigma-Aldrich, Germany) were added to a 100 ml volumetric flask. Deionized water was added to make up the volume to the mark.

### Preparation of 2% stock standards

Two millilitres of each reagent (Sigma-Aldrich) were added to a 100 ml volumetric flask. Deionized water was added to make up the volume to the mark. A stock of each of the following reagents was prepared; methanol, ethanol, 1-propanol, 2-propanol, isobutanol, 3-methyl-butanol and ethyl acetate.

### Preparation of calibration standards

A calibration standard was prepared in a range of 0.1–1.8% by diluting the stock solution with deionized water. The standards were each prepared in a 10 ml headspace vial, capped and mixed well on a vortex mixer. The standards were then immediately placed onto the headspace auto sampler tray for analysis.

### Preparation of quality controls

A 2% quality control stock solution was prepared by adding 2 ml of alcohol (Sigma-Aldrich, Germany) to a 100 ml volumetric flask and filling up to the mark with deionized water. Three quality controls at low (QC 1, 0.2%), medium (QC 2, 1.0%) and high level concentration (QC 3, 1.6%) were prepared from the stock solution. Each QC was prepared in a 10 ml headspace vial, capped with septa and aluminium crimp cap and mixed well on a vortex mixer. All 3 QCs were prepared in duplicate and positioned on the auto sampler tray for analysis after calibration standards and after every 5 duplicate samples.

### Preparation of hand sanitizer samples

#### Preparation of liquid hand sanitizer samples

In a sterile polypropylene cup (urine container) was pipetted 350 µl sanitizer to which was added 25.65 ml deionized water. Then 900 µl of this solution was transferred to a 10 ml headspace vial to which 100 µl of internal standard was also added. The vial was capped and contents mixed thoroughly on a vortex mixer before analysis.

#### Preparation of gel hand sanitizer samples

In a sterile polypropylene cup on a weighing balance 10 g of deionized water was measured and 0.350 g of gel hand sanitizer was also added. The urine container was filled up with more deionized water until a mass of 25 g was reached. The cup was capped and shaken to mix contents well. Then 900 µl of this solution was pipetted into a 10 ml headspace vial to which was also added 100 µl of internal standard. The vial was capped and contents mixed on a vortexer before analysis.

### Analysis of samples by headspace gas chromatography connected to a flame ionisation detector (HS-GC/FID)

Following sample preparation samples were immediately placed onto the Agilent G1888 headspace auto sampler tray (Agilent Technologies, USA) for analysis. The samples were analysed using a 6890N Agilent gas chromatograph (Agilent Technologies, USA) utilizing a flame ionisation detector. The column of choice was a SUPELCOWAX column (L = 30 m, ID = 0.25 mm and film thickness = 0.5 µl) purchased from Sigma-Aldrich.

### Data acquisition and processing

Quantitation was performed using the Agilent OpenLab CDS ChemStation Edition C.01.05 integration software for GC Systems, accompanying the GC system^22^. A determination coefficient (r^2^) of more than 0.999 was obtained for the calibration curves. Method accuracy, precision and repeatability were assessed by calculating the standard deviation (SD) of replicate measurements, the standard error of the mean (SEM) and the coefficient of variation (CV %) (see Tables S2–S6, in Supplementary Material).

### Data analysis

Results were analyzed using Microsoft Excel. Descriptive statistics using tables, mean and percentage was used to describe the data obtained.

## Results

**Figure 1:**
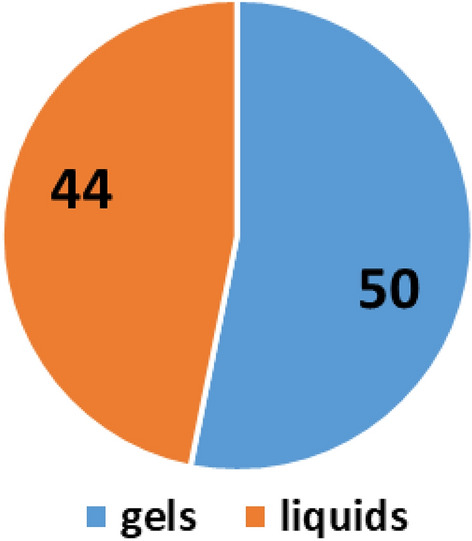
A pie chart of hand sanitizers collected around Johannesburg, comprising of gels and liquids.

Ninety-four (94) samples of hand sanitizer sold in retail stores, spaza shops and by street vendors, were randomly collected around Johannesburg during the period March to June 2020. The samples consisted of fifty (50) gels and forty-four (44) liquids as presented in Fig. 1. Forty of the sanitizers (14 liquids and 26 gels) did not have their alcohol content stated on the container and only one sample was clearly indicated as alcohol-free (Table 1). This sample set represents most of the hand sanitizer brands available and/or sold in retail stores, spaza shops and by individuals, that are in use in different households and various workplaces around Johannesburg during the CoViD-19 pandemic.

**Figure 2:**
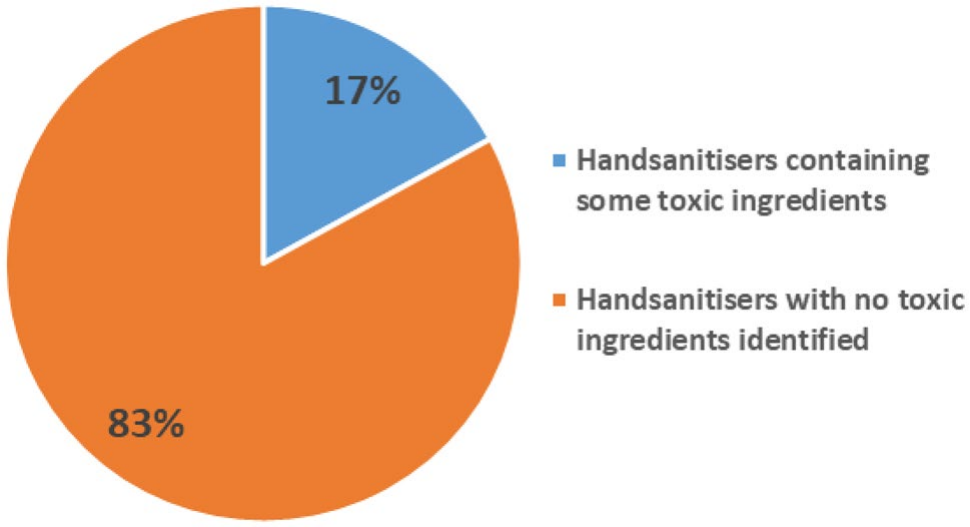
A pie chart showing a proportion of the analysed hand sanitizers found to contain toxic ingredients.

Of the 94 hand sanitizer samples collected, three sanitizer preparations were found to contain no alcohol, whereas the rest contained either ethanol or 2-propanol or a combination of these two (Table S1). Only one hand sanitizer sample contained solely 1-propanol. By comparison, liquid formulations had on average less alcohol (56.38 ± 26.74%) than the gel formulations (66.14 ± 20.95%). Of the alcohol-containing sanitizers, 37 (41%) contained less than 60% alcohol. Toxic alcohol denaturants (ethyl acetate and isobutanol) and other non-recommended alcohols (methanol and 3-methyl-butanol) were also identified in 17% of these preparations (Fig. 2).

Results from this study indicate that there are about similar number of gel hand sanitizers in existence around Johannesburg as the liquid formulations (50 gels versus 44 liquids). However, there is no certainty that this is a true representation of the then available alcohol-based hand sanitizing products as no statistical techniques were employed to collect these hand sanitizer samples. By comparison, liquid formulations (56.38 ± 27%) had on average less alcohol than the gel formulations (66.14 ± 20.95%).

To assess the quality of the results, the following criteria were examined so as to confirm the accuracy and robustness of the analytical method^23^. All peaks including ethanol and isopropanol which eluted very close to each other, had baseline separation. Peaks were sharp with narrow baseline width. The results were repeatable and reproducible as shown in Tables S2 and S3, passing the repeatability and reproducibility tests with the difference between two results chosen randomly being less than 2.83 × SD.

The average and standard deviation of the retention times for all the compounds identified were calculated. Results showed acceptable performance within run and between runs. Data for the internal standard stability and reliability are provided in Table S4. Data were generated on different days and by two analysts. There was minimal drift in retention times for all three levels of quality control throughout all the analytical runs. Linearity for the responses was assessed by examining the correlation coefficients for the calibration for all analytical runs. It is evident as shown in Table S5 that there was strong positive association (linear response) between the concentrations and the signal responses for all the analytes. Furthermore, the deviations in linearity between the analytical runs were minimal with all correlations remaining at 99% positive linear association.

Two quality controls of each of the three levels were run immediately after calibration and thereafter after every 10 samples and at the end of the analytical run. Box and Whisker plots were used to identify and remove all outliers in the data sets. Results for the quality controls were acceptable as can be seen in Table S6. Average recoveries (Av Recovery %) were acceptable. As this was a non-standard in-house developed method, Guidelines for Standard Method Performance Requirements AOAC Official Methods of Analysis^24^ was used to estimate acceptable recoveries. The recoveries were deemed acceptable for this work.

## Discussion

While more (56%) brands of hand sanitizer in this study contained the recommended concentration of alcohol, there were also many (44%) substandard and possibly subpotent preparations. Unfortunately, tests to determine if any of the analyzed hand sanitizers with lower alcohol content than is recommended had any of the virucidal activity enhancing ingredients, such as acids, were beyond the scope of this study. The study also found that only 30% (10 gels and 9 liquids) of the analyzed hand sanitizers contained ≥ 80% alcohol. Even though alcohol concentrations higher than 80% are known to be less potent against bacteria because proteins are not easily denatured in the absence of water, this bodes well for disinfection against SARS-CoV-2 as ethanol at ≥ 80% is highly effective against enveloped viruses^19^. Moreover, it was found that some hand sanitizers contained, in addition to the acceptable alcohols (ethanol, 1-propanol and 2-propanol), some toxic ingredients, such as ethyl acetate, 3-Methyl-1-butanol and methanol. This is worrying because even if a hand sanitizer contains enough alcohol as recommended or contains ingredients that enhance its virucidal activity in case of low alcohol content (< 60%), the presence of toxic ingredients renders the preparation harmful and unfit for human use. It is for this reason that it is recommended that all consumers (workplaces and the public in general) be aware of untrustworthy brands of hand sanitizer supplying substandard and possibly sub-potent sanitizer preparations or sanitizers with toxic ingredients.

The tendency for unscrupulous manufacturers in South Africa is to mislead the public by labelling their products as “SABS Approved” yet not carrying the SABS Mark Scheme number. The SABS provides on its website the information that must be available on every container of approved hand sanitizer sold in South Africa^21^. Unknowingly, using a hand sanitizer with no virucidal activity against SARS-CoV-2 may give one a false sense of security, while those using sanitizers containing toxic ingredients are likely to suffer from the associated risks. For example, exposure to methanol through both ingestion and transdermal absorption, if left untreated, can be extremely dangerous, leading to significant disability and death^25^. Even if toxic substances are just traces, the typical frequent use of hand sanitizer products throughout the day can result in very high total exposure with consequent adverse health effects.

The US FDA is therefore continually adding certain hand sanitizers found to contain toxic ingredients to import alerts, to stop these products from legally entering the U.S. market^26^, while the South African Bureau of Standards (SABS) has also warned consumers about some unscrupulous manufacturers that are making false claims that their products are SABS-approved^27^.

## Conclusion

Just like several other countries around the world, South Africa (SA) has relaxed legislation to make it easier for local businesses to rapidly produce alcohol-based hand sanitizers to meet the great surge in demand for hand sanitization products during the SARS-CoV-2 outbreak. However, those producing hand sanitizers are still advised to follow both the WHO and SABS guidelines, and avoid using poor alcohol quality which is likely to contain toxic substances. The SA public is also advised to remain alert to media reports that continually keep surfacing about hand sanitizer brands in violation of the SABS guidelines^21^, by producing sanitizer preparations that are subpotent or contain toxic substances.

It is also worth noting that the presence or addition of other pharmaceutical ingredients (e.g., chlorhexidine, triclosan, iodine/iodophores and benzalkonium chloride) may assist in instances where the alcohol-based hand sanitizers may fall short against certain bacteria and viruses^11,12^. However, though the presence of these other ingredients may impart additional antiviral and antimicrobial properties to the alcohol-based hand sanitizers, they may as well possibly exhibit some toxicity to humans. For example, although iodine is effective against most viruses and bacteria, it is also believed to cause skin irritation and discolouring, thus its presence may be harmful to humans^12,18^.

## Supplementary Information

Supplementary Tables.

## Data Availability

The datasets used and/or analysed during the current study are available from the corresponding author upon reasonable request.
