# Supplementary material for: Assessment of quality of alcohol-based hand sanitizers used in Johannesburg area during the CoViD‐19 pandemic
Source: Sci Rep. 2022 Mar 10;12:4231. doi: 10.1038/s41598-022-08117-z (PMC8908948; doi:10.1038/s41598-022-08117-z)
Supplement: Supplementary file 1 — Supplementary Tables. [file 41598_2022_8117_MOESM1_ESM.pdf]

Table S1: Alcohol content of hand sanitizers collected around Johannesburg during the CoViD-19 pandemic.

Sample Code	Liquid/ Gel/ Aerosol	% Alcohol stated on container	Sanitizer Constituents (v/v %)								
			Methanol	Ethyl acetate	2-Propanol	Ethanol	1-Propanol	Isobutanol	3-Methyl-butanol	Total Alcohol	Total Ethanol + 2-Propanol
**HS 1**	Liquid	70	-	-	0,59	75,64	-	-	-	76,22	76,22
**HS 3**	Liquid	*n/s	1,67	-	78,18	6,14	-	-	-	85,99	84,32
**HS 4**	Aerosol	40	-	-	44,87	1,26	-	-	-	46,12	46,12
**HS 8**	Liquid	70	-	-	67,55	-	-	-	-	67,55	67,55
**HS 10**	Liquid	70	-	-	69,90	-	-	-	-	69,90	69,90
**HS 18**	Liquid	74	-	-	55,40	10,94	-	-	-	66,34	66,34
**HS 21**	Liquid	70	3,92	-	3,47	48,89	-	-	2,00	58,28	52,36
**HS 24**	Liquid	95	-	-	2,43	95,29	-	-	-	97,72	97,72
**HS 29**	Liquid	70	-	-	5,08	44,86	-	-	-	49,94	49,94
**HS 33**	Aerosol	70	3,95	-	19,10	16,12	-	-	-	39,18	35,23
**HS 34**	Liquid	70	-	-	-	68,49	-	-	-	68,49	68,49
**HS 35**	Liquid	n/s	-	-	41,78	1,83	-	-	-	43,61	43,61
**HS 36**	Aerosol	62	-	-	-	32,00	-	-	-	32,00	32,00
**HS 37**	Liquid	70	-	-	-	58,58	-	-	-	58,58	58,58
**HS 38**	Liquid	70	-	-	-	80,40	-	-	-	80,40	80,40
**HS 40**	Liquid	70	-	-	-	58,07	-	-	-	58,07	58,07
**HS 41**	Aerosol	70	-	-	61,09	7,20	-	-	-	68,29	68,29
**HS 42**	Liquid	n/s	-	-	-	-	-	-	-	0,00	0,00
**HS 48**	Liquid	70	-	-	-	-	59,58	-	-	59,58	0,00
**HS 49**	Liquid	70	-	-	-	72,15	-	-	-	72,15	72,15
**HS 50**	Liquid	n/s	-	-	-	85,49	-	-	-	85,49	85,49

Sample Code	Liquid/ Gel/ Aerosol	% Alcohol stated on container	Sanitizer Constituents (v/v %)								
			Methanol	Ethyl acetate	2-Propanol	Ethanol	1-Propanol	Isobutanol	3-Methyl-butanol	Total Alcohol	Total Ethanol + 2-Propanol
**HS 51**	Liquid	70	-	-	-	83,51	-	-	-	83,51	83,51
**HS 52**	Liquid	n/s	-	-	48,82	-	-	-	-	48,82	48,82
**HS 55**	Liquid	70	-	-	74,36	6,18	-	-	-	80,55	80,55
**HS 58**	Aerosol	70	-	-	-	65,84	-	-	-	65,84	65,84
**HS 59**	Liquid	70	-	-	-	51,15	-	-	-	51,15	51,15
**HS 61**	Liquid	72	-	-	-	70,42	-	-	-	70,42	70,42
**HS 64**	Liquid	60	-	-	-	75,36	-	-	-	75,36	75,36
**HS 65**	Liquid	70	-	-	-	71,94	-	-	-	71,94	71,94
**HS 67**	Liquid	n/s	-	-	5,30	63,32	1,68	2,33	1,70	74,34	68,63
**HS 68**	Liquid	n/s	5,55	-	-	54,05	-	1,15	0,80	61,55	54,05
**HS 71**	Liquid	70	-	-	4,74	53,30	-	-	-	58,04	58,04
**HS 72**	Aerosol	70	-	-	4,03	63,67	-	-	-	67,70	67,70
**HS 73**	Liquid	75	-	-	2,73	16,99	58,75	-	-	78,47	19,72
**HS 74**	Aerosol	n/s	-	-	-	-	-	-	-	0,00	0,00
**HS 78**	Liquid	n/s	-	-	-	-	-	-	-	0,00	0,00
**HS 79**	Liquid	n/s	-	-	2,15	19,50	-	-	-	21,65	21,65
**HS 81**	Aerosol	70	-	-	4,31	61,75	6,20	2,40	1,82	76,48	66,06
**HS 83**	Liquid	75	-	-	-	59,60	1,39	6,24	0,12	67,36	59,60
**HS 84**	Liquid	n/s	-	-	-	20,44	-	-	-	20,44	20,44
**HS 88**	Aerosol	80	-	-	2,06	82,04	-	-	-	84,10	84,10
**HS 91**	Liquid	n/s	1,50	-	-	16,67	-	-	-	18,17	16,67
**HS 93**	Liquid	n/s	-	-	-	87,88	-	-	-	87,88	87,88
**HS 94**	Liquid	n/s	-	-	89,85	5,88	-	-	-	95,72	95,72
										**STDEV**	**26,74**
										**AVERAGE**	**56,38**

Sample Code	Liquid/ Gel/ Aerosol	% Alcohol stated on container	Sanitizer Constituents (v/v %)								
			Methanol	Ethyl acetate	2-Propanol	Ethanol	1-Propanol	Isobutanol	3-Methyl-butanol	Total Alcohol	Total Ethanol + 2-Propanol
**HS 2**	Gel	40	-	-	-	53,48	-	-	-	53,48	53,48
**HS 5**	Gel	n/s	-	-	-	64,17	-	-	-	64,17	64,17
**HS 6**	Gel	70	-	-	-	59,55	-	-	-	59,55	59,55
**HS 7**	Gel	n/s	-	-	53,90	2,11	-	-	-	56,01	56,01
**HS 9**	Gel	70	-	-	73,46	-	-	-	-	73,46	73,46
**HS 11**	Gel	70	-	-	59,37	4,06	8,81	-	-	72,23	63,43
**HS 12**	Gel	n/s	-	-	-	88,16	-	-	-	88,16	88,16
**HS 13**	Gel	n/s	-	-	-	70,75	-	-	-	70,75	70,75
**HS 14**	Gel	70	-	-	-	81,14	-	-	-	81,14	81,14
**HS 15**	Gel	n/s	-	-	-	67,05	-	-	-	67,05	67,05
**HS 16**	Gel	63	-	-	-	68,83	-	-	-	68,83	68,83
**HS 17**	Gel	n/s	-	-	-	73,89	-	-	-	73,89	73,89
**HS 19**	Gel	70	-	-	56,73	2,18	-	-	-	58,90	58,90
**HS 20**	Gel	n/s	-	-	3,31	17,61	8,04	3,31	-	32,26	20,92
**HS 22**	Gel	72	-	-	-	67,56	-	-	-	67,56	67,56
**HS 23**	Gel	65	-	-	-	65,00	-	-	-	65,00	65,00
**HS 25**	Gel	n/s	-	-	-	85,10	-	-	-	85,10	85,10
**HS 26**	Gel	n/s	-	-	-	90,32	-	-	-	90,32	90,32
**HS 27**	Gel	n/s	-	-	-	61,18	-	-	-	61,18	61,18
**HS 28**	Gel	n/s	3,04	16,04	2,15	45,97	5,61	2,07	1,44	76,32	48,12
**HS 30**	Gel	70	-	-	14,76	29,49	-	-	-	44,25	44,25
**HS 31**	Gel	65	-	-	-	66,53	-	-	-	66,53	66,53
**HS 32**	Gel	n/s	-	-	-	78,48	-	-	-	78,48	78,48
**HS 39**	Gel	n/s	-	-	-	85,12	-	-	-	85,12	85,12

Sample Code	Liquid/ Gel/ Aerosol	% Alcohol stated on container	Sanitizer Constituents (v/v %)								
			Methanol	Ethyl acetate	2-Propanol	Ethanol	1-Propanol	Isobutanol	3-Methyl-butanol	Total Alcohol	Total Ethanol + 2-Propanol
**HS 43**	Gel	71	1,75	-	1,16	69,51	-	-	-	72,42	70,67
**HS 44**	Gel	62	-	-	-	67,82	-	-	-	67,82	67,82
**HS 45**	Gel	70	-	-	52,61	-	-	-	-	52,61	52,61
**HS 46**	Gel	n/s	1,16			74,39				75,55	74,39
**HS 47**	Gel	62	-	-	0,33	55,06	-	-	-	55,39	55,39
**HS 53**	Gel	n/s	-	-	-	2,55	47,09	-	-	49,65	2,55
**HS 54**	Gel	n/s	-	-	-	98,65	-	-	-	98,65	98,65
**HS 56**	Gel	70	-	-	-	79,42	-	-	-	79,42	79,42
**HS 57**	Gel	n/s	-	-	-	75,04	-	-	-	75,04	75,04
**HS 60**	Gel	n/s	-	-	-	61,22	-	-	-	61,22	61,22
**HS 62**	Gel	70	-	-	0,30	69,22	-	-	-	69,51	69,51
**HS 63**	Gel	alcohol-free	-	-	-	0,51	-	-	-	0,51	0,51
**HS 66**	Gel	70	1,98	-	-	75,72	-	-	-	77,69	75,72
**HS 69**	Gel	n/s	-	-	-	58,18	-	-	-	58,18	58,18
**HS 70**	Gel	70	-	-	-	69,24	-	-	-	69,24	69,24
**HS 75**	Gel	n/s	-	-	-	69,39	-	-	-	69,39	69,39
**HS 76**	Gel	70	-	-	-	62,87	-	-	-	62,87	62,87
**HS 77**	Gel	n/s	-	-	-	68,12	-	-	-	68,12	68,12
**HS 80**	Gel	75	-	-	1,37	67,21	-	-	-	68,58	68,58
**HS 82**	Gel	70	2,00	-	0,93	61,77	-	-	-	64,70	62,70
**HS 85**	Gel	n/s	-	-	-	99,18	-	-	-	99,18	99,18
**HS 86**	Gel	n/s	-	-	-	88,40	-	-	-	88,40	88,40
**HS 87**	Gel	n/s				90,90				90,90	90,90
**HS 89**	Gel	70	-	-	64,83	4,15	-	-	-	68,98	68,98
**HS 90**	Gel	n/s	-	-	-	97,49	-	-	-	97,49	97,49

Sample Code	Liquid/ Gel/ Aerosol	% Alcohol stated on container	Sanitizer Constituents (v/v %)								
			Methanol	Ethyl acetate	2-Propanol	Ethanol	1-Propanol	Isobutanol	3-Methyl-butanol	Total Alcohol	Total Ethanol + 2-Propanol
**HS 92**	Gel	70	-	-	0,30	69,22	-	-	-	69,51	69,51
										**STD DEV**	**20,95**
										**AVERAGE**	**66,14**
***n/s: not stated**											

Table S2 : Assessment for Repeatability (26).

QC1 (0,20%)				
	SD_r	RSD_r	$Difference_{2\ results}$	$Difference_{2\ results}$ < RSD_r
MeOH	0,087	0,245	0,142	✓
EthAcet	0,033	0,092	0,025	✓
Isop	0,033	0,093	0,081	✓
EtOH	0,029	0,082	0,071	✓
1-prop	0,046	0,130	0,074	✓
Isobut	0,043	0,122	0,083	✓
3-Mebut	0,042	0,120	0,069	✓
QC2 (1,00%)				
MeOH	0,178	0,503	0,062	✓
EthAcet	0,186	0,526	0,039	✓
Isop	0,142	0,402	0,001	✓
EtOH	0,118	0,334	0,000	✓
1-prop	0,138	0,390	0,032	✓
Isobut	0,142	0,401	0,014	✓
3-Mebut	0,160	0,454	0,164	✓
QC3 (1,60%)				
MeOH	0,172	0,486	0,425	✓
EthAcet	0,268	0,757	0,041	✓
Isop	0,158	0,447	0,083	✓
EtOH	0,171	0,485	0,432	✓
1-prop	0,172	0,488	0,129	✓
Isobut	0,171	0,485	0,113	✓
3-Mebut	0,226	0,641	0,402	✓

SD_r – Repeatability standard deviation

$RSD_r = 2.83 \times SD_r$

✓ - Acceptable

MeOH-Methanol, EthAcet – Ethyl Acetate, Isop – Isopropanol, EtOH – Ethanol, 1-prop – 1-Propanol, Isobut – Isobutanol, 3-Mebut – 3-Methybutanol

Table S3: Assessment for Reproducibility (26).

	SD_R	RSD_R	$Difference_{2\ results}$	$Difference_{2\ results} < RSD_r$
QC1 (0,20%)				
MeOH	0,060	0,169	0,095	✓
EthAcet	0,031	0,089	0,051	✓
Isop	0,035	0,099	0,067	✓
EtOH	0,019	0,055	0,001	✓
1-prop	0,041	0,116	0,059	✓
Isobut	0,052	0,146	0,072	✓
3-Mebut	0,043	0,123	0,034	✓
QC2 (1,00%)				
MeOH	0,074	0,211	0,007	✓
EthAcet	0,072	0,203	0,132	✓
Isop	0,073	0,207	0,171	✓
EtOH	0,092	0,259	0,161	✓
1-prop	0,073	0,208	0,124	✓
Isobut	0,068	0,191	0,064	✓
3-Mebut	0,054	0,153	0,040	✓
QC3 (1,60%)				
MeOH	0,150	0,425	0,353	✓
EthAcet	0,113	0,318	0,102	✓
Isop	0,065	0,184	0,058	✓
EtOH	0,072	0,204	0,006	✓
1-prop	0,075	0,212	0,042	✓
Isobut	0,078	0,221	0,023	✓
3-Mebut	0,102	0,290	0,183	✓

SD_R : Reproducibility standard deviation

$RSD_R = 2.83 \times SD_R$

Table S4 : Retention Time parameters for Quality Control and Internal Standard.

		Peak retention Time (min)							
		Acet	MeOH	EthAcet	Isop	ETOH	I-prop	Isobut	3-Mebut
QC 1 (0,20%)	Average	2,18	3,94	4,28	4,92	5,04	7,12	8,13	10,34
	min	2,16	3,87	4,21	4,85	4,97	7,07	8,07	10,31
	max	2,21	4,01	4,34	4,98	5,10	7,16	8,27	10,44
	SD	0,013	0,034	0,035	0,031	0,030	0,023	0,049	0,023
QC 2 (1,00%)	Average	2,18	3,97	4,24	4,92	5,03	7,12	8,12	10,34
	min	2,16	3,90	4,18	4,87	4,98	7,08	8,08	10,31
	max	2,21	4,03	4,31	4,98	5,09	7,16	8,24	10,36
	SD	0,013	0,033	0,032	0,031	0,030	0,022	0,027	0,013
QC 3 (1,60%)	Average	2,18	3,97	4,23	4,92	5,03	7,12	8,12	10,34
	min	2,16	3,91	4,17	4,87	4,97	7,08	8,09	10,21
	max	2,21	4,04	4,30	4,98	5,09	7,16	8,15	10,36
	SD	0,013	0,035	0,034	0,032	0,031	0,022	0,018	0,021

Table S5: Correlation coefficient for calibrations

	Correlation R						
Date	MeOH	EthAcet	Isop	EtOH	1-prop	Isobut	3-Mebut
14.08.2020	0,99356	0,99061	0,99319	0,99482	0,99202	0,99255	0,99455
18.08.2020	0,99836	0,99489	0,99853	0,99838	0,99687	0,99641	0,99692
27.08.2020	0,99412	0,99319	0,99548	0,99270	0,99204	0,99150	0,99439
09.09.2020	0,99337	0,99217	0,99202	0,99327	0,99184	0,99317	0,99430
16.09.2020	0,99743	0,99904	0,99922	0,99951	0,99901	0,99901	0,99752
21.09.2020	0,99181	0,99792	0,99575	0,99721	0,99458	0,99307	0,99075

Average	0,99478	0,99464	0,99570	0,99598	0,99439	0,99429	0,99474
min	0,99181	0,99061	0,99202	0,99270	0,99184	0,99150	0,99075
max	0,99836	0,99904	0,99922	0,99951	0,99901	0,99901	0,99752
SD	0,00255	0,00331	0,00284	0,00280	0,00301	0,00284	0,00240

Table S6 : Quality Control parameters

		n	Mean	Av Recovery%	SD	max	min
QC1 (0,20%)	MeOH	56	0,21	104	0,06	0,35	0,11
	EthAcet	53	0,21	107	0,04	0,32	0,12
	Isop	55	0,25	127	0,04	0,33	0,20
	EtOH	52	0,23	114	0,03	0,30	0,17
	1-prop	55	0,28	140	0,05	0,40	0,19
	Isobut	56	0,29	144	0,05	0,42	0,18
	3-Mebut	54	0,26	128	0,05	0,41	0,18
QC2 (1,00%)	MeOH	52	0,92	92	0,13	1,23	0,61
	EthAcet	56	0,95	95	0,23	1,53	0,54
	Isop	56	0,92	92	0,17	1,25	0,59
	EtOH	56	0,87	87	0,14	1,13	0,65
	1-prop	56	0,91	91	0,17	1,19	0,57
	Isobut	56	0,89	89	0,17	1,18	0,55
	3-Mebut	56	0,91	91	0,17	1,20	0,57
QC3 (1,60%)	MeOH	54	1,42	89	0,16	1,83	1,08
	EthAcet	57	1,57	98	0,23	2,04	1,02
	Isop	57	1,47	92	0,19	1,85	1,02
	EtOH	57	1,46	91	0,17	1,78	1,04
	1-prop	57	1,50	93	0,21	1,79	0,97
	Isobut	57	1,44	90	0,22	1,76	0,94
	3-Mebut	56	1,41	88	0,25	1,84	0,92
